# Enhancing diagnostic accuracy of multiple myeloma through ML-driven analysis of hematological slides: new dataset and identification model to support hematologists

**DOI:** 10.1038/s41598-024-61420-9

**Published:** 2024-05-15

**Authors:** Caio L. B. Andrade, Marcos V. Ferreira, Brenno M. Alencar, Ariel M. A. Junior, Tiago J. S. Lopes, Allan S. dos Santos, Mariane M. dos Santos, Maria I. C. S. Silva, Izabela M. D. R. P. Rosa, Jorge L. S. B. Filho, Matheus A. Guimaraes, Gilson C. de Carvalho, Herbert H. M. Santos, Márcia M. L. Santos, Roberto Meyer, Tatiane N. Rios, Ricardo A. Rios, Songeli M. Freire

**Affiliations:** 1 Institute of Health Sciences, Federal University of Bahia, Salvador, 40110-902 Brazil; 2 Institute of Computing, Federal University of Bahia, Salvador, 40170-110 Brazil; 3 Nezu Life Sciences, 76149 Karlsruhe, Germany; 4 Hospital Universitario Professor Edgard Santos - HUPES, Federal University of Bahia, Salvador, 40110-902 Brazil

**Keywords:** Myeloma, Machine learning

## Abstract

Multiple Myeloma (MM) is a hematological malignancy characterized by the clonal proliferation of plasma cells within the bone marrow. Diagnosing MM presents considerable challenges, involving the identification of plasma cells in cytology examinations on hematological slides. At present, this is still a time-consuming manual task and has high labor costs. These challenges have adverse implications, which rely heavily on medical professionals’ expertise and experience. To tackle these challenges, we present an investigation using Artificial Intelligence, specifically a Machine Learning analysis of hematological slides with a Deep Neural Network (DNN), to support specialists during the process of diagnosing MM. In this sense, the contribution of this study is twofold: in addition to the trained model to diagnose MM, we also make available to the community a fully-curated hematological slide dataset with thousands of images of plasma cells. Taken together, the setup we established here is a framework that researchers and hospitals with limited resources can promptly use. Our contributions provide practical results that have been directly applied in the public health system in Brazil. Given the open-source nature of the project, we anticipate it will be used and extended to diagnose other malignancies.

## Introduction

Multiple Myeloma (MM) is a plasma cell oncohematology with the highest incidence, being more frequent in individuals over 50 years old. According to the International Agency for Research on Cancer of the World Health Organization (WHO), in 2020, MM was responsible for 176,404 new cases and 117,077 deaths of patients of both sexes^1^. In Brazil, the Brazilian Oncology Panel shows that, between 2013 and 2019, around 2600 cases of MM were diagnosed annually, representing a rate of 1.24 cases/100,000 inhabitants—1.47% of new cases worldwide^2,3^. This oncology corresponds to about 1% of malignant tumors and 10–15% of hematological neoplasms^1^. The diagnosis and patient’s prognosis are based on a wide range of exams, from clinical evaluations of the patient’s condition, through biochemical, and histological tests, to more advanced techniques such as immunophenotyping and cytogenetics. Among such exams, we highlight Myelogram, which is characterized as being primarily adopted and dependent on manual procedures, implying several limitations on the speed and capacity of meeting the growing demand for new cases in contrast to the limited availability of qualified professionals^1–3^.

Plasma cells are effector cells of the B lymphocyte lineage with an essential role in the humoral immune response due to the production and secretion of antibodies. Plasma cells originate from B lymphocytes that have undergone differentiation into plasmablasts $\longrightarrow$ proplasmocytes $\longrightarrow$ plasma cells^4–6^. After being differentiated into plasma cells, their function is to produce immunoglobulins, which act in the humoral defense against viruses, bacteria, and allergens^4–6^. They present the markers CD19, CD20, CD22, and surface immunoglobulin (IgM or IgD) as immunophenotypic characteristics. When transformed into plasmocytes, they lose or decrease the expression of B lymphocyte markers and begin to express CD27, CD38, and CD138^6,7^. Their cytomorphological characteristics are 12–18 $\upmu \textrm{m}$ in diameter, rounded or oval, a meager nucleus-cytoplasm ratio, a dense chromatin nucleus, evenly distributed, regular outline, generally eccentric location, an oval or round shape, and characteristic perinuclear clear halo. Moreover, their cytoplasm is relatively abundant, deeply basophilic (intense blue), and without granules^6,7^. In summary, they are typical cells in lymph nodes and bone marrow but extremely rare in peripheral blood, except in a few cases of viruses such as rubella (but always less than 5%), in rare cases of MM in terminal stages or in the rare and aggressive primary plasma cell leukemia^6,7^.

In MM, the exacerbated proliferation of plasmocytes, resulting from a hematopoietic malignancy, leads to a high concentration of these cells in the bone marrow, thus causing, in symptomatic cases, high secretion of monoclonal protein, light or heavy chains of incomplete antibodies detectable in the blood or urine, and organ dysfunction. Symptoms of myeloma may include extreme tiredness, weakness, pallor and weight loss, malfunction of the kidneys, swelling in the legs, excessive thirst, loss of appetite, severe constipation, bone pain (especially in the spine), spontaneous fractures, and constant infections^5,6^.

The importance of leveraging Artificial Intelligence (AI) to support patients afflicted with MM has been widely emphasized in studies recently published in the literature. In 2022, researchers disseminated a review of studies devoted to applying Machine Learning (ML) and Deep Neural Networks (DNN) methods to improve the disease’s diagnosis, prognosis, and treatment selection^8^. Besides gathering related works with their respective AI-based methods and datasets, the authors conclude that the use of ML and DNN has the potential to increase our knowledge, thus providing a better understanding of the myeloma genesis mechanisms. According to the authors, adopting AI-based methods is still an open problem in this area, which may drive the decision-making processes in the near future. Since 2019, it is possible to notice an increasing amount of papers^9–17^ proposing DNN architectures (e.g., Recurrent Neural Network - RNN, and Convolutional Neural Network - CNN) to process images from Computed Tomography (CT) scanners in order to support precise and early diagnosis of MM. In such studies, the proposed DNN architectures were capable of suppressing image noises and artifacts without losing spatial resolution, thus presenting the potential to improve radiologists’ ability to visualize lesions in multiple myeloma^18^. Similarly, in 2022, authors presented a new study on using DNN to examine Magnetic Resonance Imaging (MRI) images and provide satisfactory diagnostic performance in diagnosing MM^19^, and supporting differentiate spinal MM and metastases originating from lung cancer^20^. From a different perspective, researchers have also designed DNN architectures to model images from Immunofixation Electrophoresis (IFE) tests in order to identify monoclonal proteins (M-proteins, makers for multiple myeloma) from human serum (or urine)^21^. The primary distinguishing aspect between our proposal and prior works lies in the dataset utilized for investigating MM. Rather than examining CT, MRI, or IFE images, we have devised a novel architecture that is directly trained on plasma cells. However, it is essential to highlight that their DNN architectures were carefully analyzed and inspired the design of our new model.

In relation to the process of modeling plasma cells, we have found some manuscripts strongly related to our investigation. The first one, published in 2021, presented a CNN architecture trained on a dataset with slides prepared from the bone marrow aspirate of the healthy and cancer subjects^22^. However, the images derived from this dataset differ significantly from ours, since each segmented image comprises only a single cell positioned at the origin. The second one, published in 2022, presents an approach combining DNN and a decomposition method to segment plasma cells into nuclei and membranes^23^. The dataset considered by the authors contains 298 images with high resolution.

Lastly, it is worth noting a study presented in 2022, where the researchers analyzed patients’ data to construct a Graph-based Neural Network for predicting the likelihood of developing MM^24^. Despite differences in the dataset and DNN architecture compared to our study, we incorporated this manuscript into our review owing to its relevance in addressing this disease.

In our study, we have dedicated a great effort to aggregate knowledge to the current state-of-the-art towards facilitating the discovery of effective strategies for managing and diagnosing MM. In summary, the contributions presented in this study were conducted in two phases. Initially, we generated a novel dataset with images comprising diverse cell types (e.g., Plasma cells, Lymphocytes, and Erythroblasts), which were analyzed and manually labeled by a committee of specialists. While our primary objective was to improve the diagnosis of MM by identifying Plasma cells, the comprehensive nature of the dataset provides a valuable resource for researchers investigating other cell types and diseases.

Subsequently, we conducted a rigorous analysis of the existing modeling processes described in the literature and devised a specific DNN architecture to highlight the significance of our dataset. Our DNN model has a practical emphasis on assisting specialists in rendering more accurate diagnoses to patients enrolled in the public health system, particularly since such professionals lack access to advanced technological aids for decision-making. Our proposal addresses this deficiency by leveraging the available infrastructure without incurring additional costs to acquire expensive experimental setups to capture and process high-resolution images. The dissemination of our dataset, DNN architecture, and findings extends support to MM patients in developing countries and new studies on diseases in other cell types.

## Results

### Sample preparation

The diagnosis of MM has made several advances in recent years with the implementation of new techniques, such as immunophenotyping and oncogenetic analyses. However, it still relies on the myelogram, which involves a laborious methodology, demanding significant effort and time from hematologists. Moreover, these analyses present a risk of human error for being an observer-dependent approach^25–27^. Although several studies bring new perspectives in the transition from this manual technique to an automated format using AI, several image repositories were produced with high-cost equipment, often inaccessible to laboratories that serve lower-income populations, and images from slides with higher quality than usually observed in the daily routine of hematology professionals. Furthermore, most bone marrow slide datasets identify only plasma cells, not allowing the automatic evaluation of the percentage of plasma cells^28,29^.

**Figure 1:**
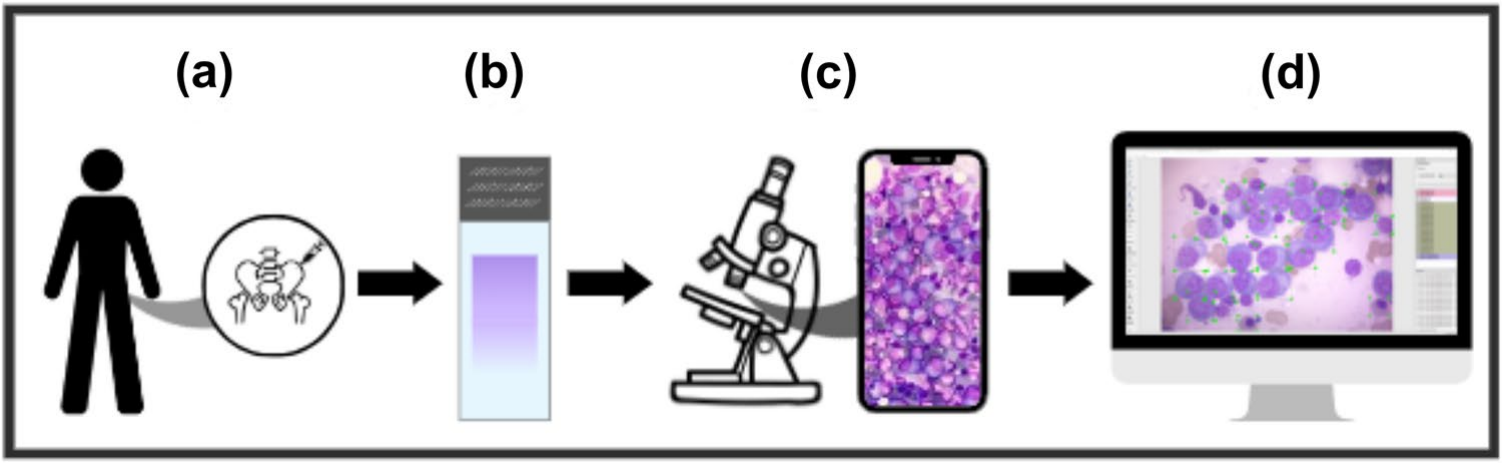
(**a**) Bone marrow aspirate procedure; (**b**) Wright–Giemsa stained bone marrow aspirate smear slides from MM patients, analyzed by the oncohematology and immunophenotyping service of the Laboratory of Immunology and Molecular Biology; (**c**) Observation of stained slides in visible light optical microscopes and image capture by smartphone device; (**d**) Identification and labeling of detected cells.

The present study includes samples of patients from the Brazilian public health system (Unified Health System) attended by the Laboratory of Immunology and Molecular Biology from the Federal Univeristy of Bahia (LabImuno/UFBA) and diagnosed with MM and other hematology diseases, as illustrated in Fig. 1a. For the construction of our image dataset, histological slides of bone marrow aspirate stained with Wright–Giemsa (SIGMA-ALDRICH, MERCK) were selected (Fig. 1b), visualized with a NIKON ECLIPSE CI visible light microscope in a 100x objective with the use of immersion oil (Fig. 1c). Each slide was positioned to view the crest region of the smear, and the nucleated cells found were photographed with a smartphone camera with the aid of a universal mobile phone support for the microscope.

Next, a committee formed by trained researchers and hematology specialists individually analyzed every captured image and manually labeled all cells according to their types (e.g., plasma cells, lymphocytes, and erythroblasts), as shown in Fig. 1d. All those specialists work in the Brazilian public health system and have vast experience in MM diagnoses. We emphasize that cells lacking unanimous agreement among the specialists were excluded from labeling and not utilized during the training phases to prevent the propagation of errors.

During this task, we used the LabelImg tool^30^, which is a graphical interface that helps users to easily draw bonding boxes on Regions of Interest (ROI) and set labels that are later used to train AI-based techniques.

Finally, the labeled images were sent to the AI specialists to train an DNN models capable of automatically identifying plasma cells. In this work, although our dataset is composed by images with different types of cells, supporting the investigation of several biological subjects, we have focused our attention to support the diagnosis of MM. Therefore, the labels of our cells were categorized into 1,891 “plasma cells” and 1,931 “non-plasma cells”.

This project fulfills scientific objectives and addresses a significant social need, making it an essential contribution. The LabImuno/UFBA laboratory is a leading scientific facility that offers diagnoses for various hematological conditions. Analyzing blood samples supports the public healthcare system in the state of Bahia, Brazil. As a result, the outcomes presented in this manuscript have immediate practical implications, emphasizing the importance of this project as a crucial step towards advancing healthcare practices and improving the life quality of local patients.

### Machine learning framework

The objective of the present study is to investigate the automatic identification of plasma cells in images through the development of an ML-based approach. The primary challenge lies in devising an approach that aids specialists in the diagnosis of MM. Notably, our work is distinct from usual approaches, focusing on modeling images captured using standard smartphone cameras. This feature is particularly pertinent as it facilitates the decision-making process in hospitals with resource-constrained settings.

The experimental setup designed to build our plasma-cell classifier was based on traditional steps defined by the open standard process model Cross-Industry Standard Process for Data Mining (CRISP-DM)^31^. This process is extensively recognized in Machine Learning tasks, and our project encapsulates three main steps: (i) Data understanding and preparation, a.k.a. preprocessing; (ii) Modeling; and (iii) Evaluation. During the preprocessing step, as mentioned in Section “Sample Preparation”, the dataset was created using histological slides of bone marrow aspirate stained with Wright-Giemsa. Next, a committee formed by specialists who work in the hematology service of the health system, two hematologists and two biomedics with specialization in hematology, was responsible for individually drawing bounding boxes on regions of interest and explicitly defining labels to inform the cell types. The final cell labels were set after a consensus among all specialists.

Subsequently, the modeling process was executed by employing a stratified 10-fold cross-validation strategy. The optimal outcomes were determined based on the mean identification performance across all folds. This approach was adopted to mitigate the possibility of drawing conclusions by chance and finding the best performance of the model. Further information on the modeling and evaluation steps is provided in Section “Material and methods”.

### Plasma cell identification

Although the dataset presented in this manuscript was initially curated by specialists to identify diverse types of cells, we have devoted our effort to creating an DNN-based model capable of detecting plasma cells and, consequently, supporting the diagnosis process.

**Figure 2:**
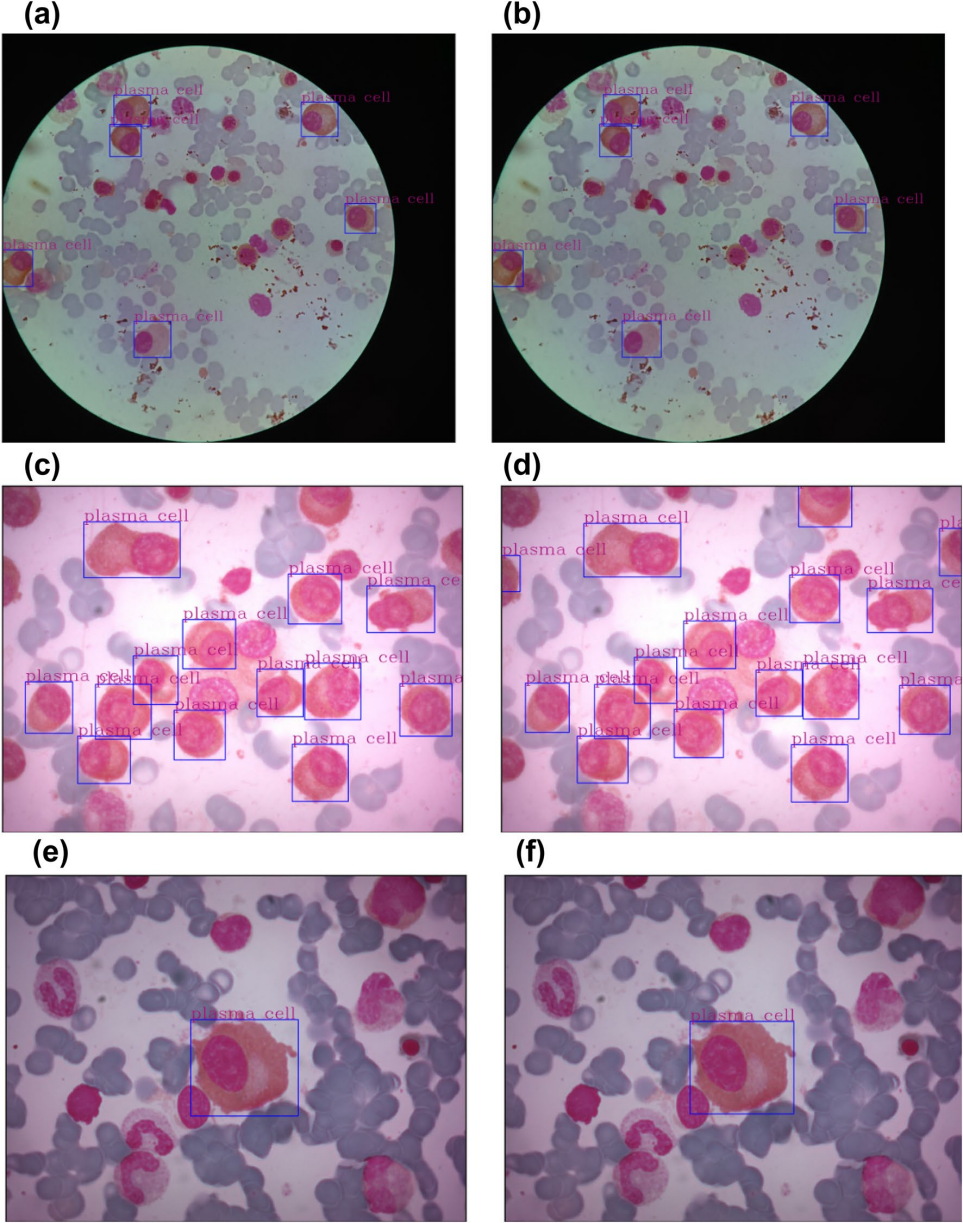
Example of expected (ground truth) and predicted bounding boxes on plasma cells. Images (**a**), (**c**), and (**e**) show the regions of interest drawn by specialists. On the other hand, Images (**b**), (**d**), and (**f**) illustrates the bounding boxes defined by our AI-based approach.

In order to better understand our investigation, consider Fig. 2, which presents a set of images used in our experimental setup. In the first column, Fig. 2a, c and e show images labeled by specialists. On the other hand, images in the second column (b, d, and f) show labels provided by our approach. Such images illustrate the challenging task of identifying plasma cells on different image backgrounds. Moreover, one may notice that images present different cells and their residues, which affect the learning process. Another important observation is the impact on the final evaluation. By considering the expected (Fig. 2a,e) and predicted (Fig. 2b,f) images, one may see a perfect alignment with more precise bounding boxes (less background). However, Fig. 2c and d show a significant agreement (true positive cases) between expected and predicted bounding boxes, but with some relevant differences. By looking at these figures, there are plasma cells not classified by the specialists but detected by our models. Although the bounding boxes identified by our models are probably plasma cells, specialists have not highlighted them due to a confidence issue, i.e., once the cells are not wholly visually seen, they are not considered by the specialists. In this case, the detections performed by our model are considered false positives. The considered “misclassification” increases the general error, even partially detecting plasma cells.

After visually inspecting our approach, we have performed an experimental evaluation using stratified 10-fold cross-validation (10 executions with different combinations of training and validation), employing metrics conventionally utilized in object identification tasks, as shown in Table 1. To summarize all metrics, in the last row, we show the mean values per metric calculated on all folds. The mean results emphasize the performance of our approach in terms of exactitude and completeness between 80 and 84% and mean F1-scores greater than 80%.

**Table 1:** Plasma cell identification using different evaluation metrics.

Fold	#Images	Labels	Precision	Recall	F1-score
1	53	175	0.83	0.88	0.85
2	51	155	0.85	0.76	0.80
3	52	198	0.81	0.87	0.84
4	52	218	0.75	0.53	0.62
5	50	194	0.88	0.79	0.83
6	51	188	0.84	0.93	0.88
7	49	194	0.81	0.82	0.81
8	51	204	0.79	0.76	0.78
9	50	184	0.91	0.86	0.88
10	53	181	0.93	0.85	0.88
$\mu _{\textrm{Fold}}$	–	–	0.84	0.80	0.81

Mean values computed from 10 test folds show high general performances.

**Figure 3:**
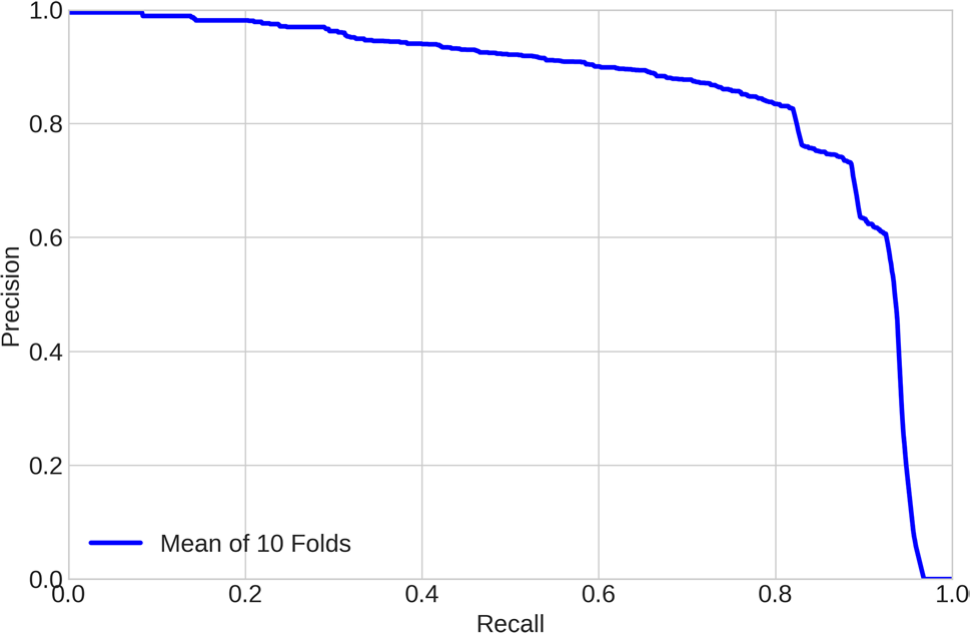
Precision-recall curve: the approximation on the right-top corner emphasizes the important performance of our approach.

Finally, Fig. 3 shows the Precision-Recall (PR) curve on all images randomly used to compose the test folds. By plotting the PR curve, we can better visualize the relationship between Precision and Recall. This is particularly useful in our scenario where the cost of misclassified regions is caused by different sources. The Precision metric takes into account false positive identifications (i.e., other cells considered plasma cells), whereas Recall measures the influence of missed plasma cells. The combination of both metrics in a single curve allows us to verify whether there is a classification bias. In summary, Precision and Recall getting close to 1.0 lead the curve to approximate to the upper-right corner, which is the best possible result. After analyzing this figure along with their individual values (Table 1), we conclude that our approach provides great individual (Precision and Recall) and combined (F1-score and Precision-Recall curve) results.

## Discussion

The present investigation conducted initial assessments to determine a viable strategy for creating an image repository that emulates the MM diagnostic protocol in Brazil, including the demarcation of non-plasma cell populations, for subsequent computation of plasma cell proportions. Consequently, the collected dataset was utilized to assess the optimal software framework for training an AI system, based on Machine Learning, to discern immune system cells, specifically plasma cells, and quantify them.

The preparation process for analyzing myelogram samples involves several stages, including processing, reading, and analyzing the samples for the release of diagnostic reports and therapeutic procedures. Although using an ML model does not eliminate the need for hematology specialists to examine myelogram images, it can simplify the verification and certification process, making it more accessible, precise (once our contribution can assist specialists by highlighting cells in advance), and rapid while also considering the total and proportional count of plasma cells in the smear. Implementing a tested and validated ML model can support hematologists specializing in myelograms, ensuring safety and quality. With reduced slide reading time, hematology specialists can attend to other critical technical and medical functions required for patient care.

Specifically, our model has demonstrated to be a valuable tool in supporting the diagnosis of Myeloma, a traditionally time-consuming and subjective task. By analyzing large datasets of patient data, ML algorithms can identify patterns and features that distinguish myeloma from other forms of cancer, resulting in more accurate and efficient diagnosis. Additionally, the use of ML in the classification of myeloma vs. non-myeloma represents a critical step towards improving cancer diagnosis and treatment, with the potential to impact patient outcomes and advance our understanding of the disease.

The myelogram procedure plays a pivotal role in the initial diagnosis of oncohematological disorders, yet it presents a significant obstacle to rapid and safe diagnosis. While several automated systems are available for sample preparation in hematology, bone marrow preparation for myelogram remains a labor-intensive manual process. Following bone marrow aspiration, hematologists must create smears of the samples on microscope slides and stain them with Wright–Giemsa^6^. This manual procedure introduces a range of potential variations in slide quality, which the present dataset seeks to incorporate to enhance the development of AI in this field. By addressing the issue of manual slide preparation, the dataset aims to improve the accuracy and efficiency of AI models in diagnosing oncohematological disorders.

Moreover, the available datasets utilized in prior studies were generated using high-quality imaging equipment attached to microscopes. In contrast, the present dataset was produced using equipment commonly available in laboratories that cater to the Brazilian Public Health System^22,23,28,32^. From this standpoint, the cell-type classification provided by the current dataset enables the identification of other nucleated cells, which is a critical component of various diagnostic procedures and has recently been the focus of several studies aimed at automating this time-intensive process. Prior projects using smaller datasets have demonstrated promising results, albeit limited to plasma cell identification. However, these datasets do not furnish the necessary information for percentage counting of plasma cells, which is vital in identifying multiple myeloma^22,23,28,32^.

The present study marks a significant milestone in developing a reliable dataset for oncohematology diagnoses. By labeling non-plasmacytic cells and expanding the image bank to include additional labeled images, the study lays the foundation for more accurate and efficient diagnoses of oncohematological disorders. However, it is essential to acknowledge that the current dataset still needs to encompass a greater variety of plasma cell varieties, such as Mott cells, which require more refined ML algorithms to identify accurately. Currently, the dataset is composed of 512 images with 3822 identified cells. In our results, AI has already shown promising results in identifying cell areas and plasmocytes, even in the early stages of training. As such, the present study has the potential to advance the development of ML-based tools that can accurately diagnose and treat oncohematological disorders. The dataset will become increasingly robust with ongoing research and development, enabling more accurate diagnoses and better patient outcomes.

By creating a dataset that closely resembles the diagnostic routine of hematology professionals in Brazil, the study enables the development of ML models that can better meet the needs and demands of this sector. Moreover, the dataset is produced using low-cost devices that are easily adaptable to Brazil’s existing structure of diagnostic laboratories. This dataset makes it possible to implement AI-based diagnostic tools in a cost-effective and accessible manner, ultimately improving the quality and speed of diagnoses for patients with oncohematological disorders. By leveraging the unique characteristics of the Brazilian healthcare system, the present study can potentially transform how oncohematology is diagnosed and treated, both in Brazil and beyond. The remarkable outcomes achieved in plasma cell identification on our dataset pave new avenues for enhancing the diagnosis of MM. Based on our experience, adopting our final AI model can leverage basic setups, which include a microscope and smartphone camera, to support the decision-making process. Our final model is also freely accessible, enabling users to refine it by training it appropriately with specific MM images. Furthermore, we highlight the utility of our dataset, which includes labels for different cell types, in augmenting other datasets.

In oncological diagnosis, time and sensitivity are critical factors, significantly impacting the chances of successfully treating the patient. Therefore, there is a pressing need for advancements in the automation of diagnostic approaches, particularly with the growing number of diseases such as MM cases. The use of AI, trained with a dataset produced by low-cost equipment, represents a significant step towards democratizing access to healthcare. This approach aims to support specialists by underlining the identification of cells crucial for the diagnostic process. We emphasize that specialists are actively involved in this process, leveraging their expertise to determine the relevance of specific cells. In several contexts, a limited number of specialists analyzing several slides can delay diagnoses and induce errors. However, by prioritizing the recognition of pertinent cells, our contribution is essential to support the diagnostic without the need for costly equipment. By leveraging the power of AI, the diagnosis of oncohematological disorders can be made more efficient, accurate, and cost-effective. This improvement, in turn, can potentially improve patient outcomes and increase the accessibility of healthcare services, particularly in resource-limited settings. Overall, the use of AI in the diagnosis of oncohematological disorders is a critical tool in the fight against these devastating diseases. In that sense, our methodology can be extended to other malignancies, where automated classification and quantification of malignant cells can aid in early diagnosis and selecting appropriate treatments. The availability of such technology, which can be easily adapted to existing laboratory infrastructure, can revolutionize cancer diagnosis, making it more accessible, accurate, and efficient for healthcare providers and patients.

Finally, considering the constraints inherent in our study, we have identified important pathways for further research stemming from our findings, offering valuable insights for advancing the diagnosis of multiple myeloma (MM). Specifically, we outline the prospect of exploring alternative deep neural network (DNN) architectures, such as attention mechanisms and generative models, to enhance diagnostic accuracy. From a biological perspective, we intend to design new approaches and models for detecting plasma cells with multiple nuclei and structural issues relevant to understanding how MM evolves and affects the patient’s condition.

## Material and methods

### Deep neural network

The DNN architecture considered to detect plasma cells in our new dataset is based on YOLO (You Only Look Once)^33^, which is the state-of-the-art technique to detect objects in real time. We have used the YOLO version 7 that combines the highest precision along with the lowest inference time.

In summary, YOLO is based on three specific components: backbone, head, and neck. Backbone is responsible for extracting features from low to high levels in images. Next, such features are transmitted to the head component throughout the neck component, which speeds up the processing task by combining different scales and reducing the dimensionality.

The head component is in charge of detecting objects, drawing bounding boxes, and inferring the estimated classes (labels). Historically, the backbone component used different architectures: Darknet in the first version^34^, Cross-Stage Partial Network (CSP) in Version 5^35^, and Extended Efficient Layer Aggregation Network (E-ELAN) in the last version^36^. The main advantage of using E-ELAN is during the training phase, in which the gradient update is continuously improved to extract the best performance of the network. Another significant contribution of this version (v.7) is the model scaling, which allows to adjust the network on different devices and applications. By taking advantage of this functionality, we were able to configure and tune a DNN specifically devoted to identifying plasma cells and non-plasma cells in stained bone marrow aspirate smear slides. The process used to perform such a configuration was based on the traditional construction of DNN by adding and removing layers, changing image resolution, modifying channels and filters, and tuning parameters. It is also relevant to mention that YOLO v.7 uses an optimization process based on re-parameterization planning (RP)^37^.

Aiming to reach the best performance possible, we have used an innovative approach referred to as transfer learning to accelerate the training process. In summary, this approach allowed us to re-use the knowledge learned from previous tasks to boost the performance of detecting plasma cells. In this sense, we have used frozen weights and parameters, especially from the backbone and neck layers, which were initially modeled on the dataset Microsoft COCO (Common Object in Context)^38^. COCO is a large-scale object detection, segmentation, and captioning dataset containing several images traditionally used to perform different tasks in Computer Vision. Next, we performed a fine-tuning process on our images, significantly increasing our results in detecting bounding box sizes, probabilities, and classifications of plasma cells.

The final configuration of our training process was obtained after using a batch with a size equal to 32, 300 epochs, 64 GB of GPU memory, a learning rate equal to 0.01, and an Adam optimizer. After using our images during the training phase, the resultant model presented 362 layers with 70,782,444 parameters.

### Evaluation metrics

**Figure 4:**
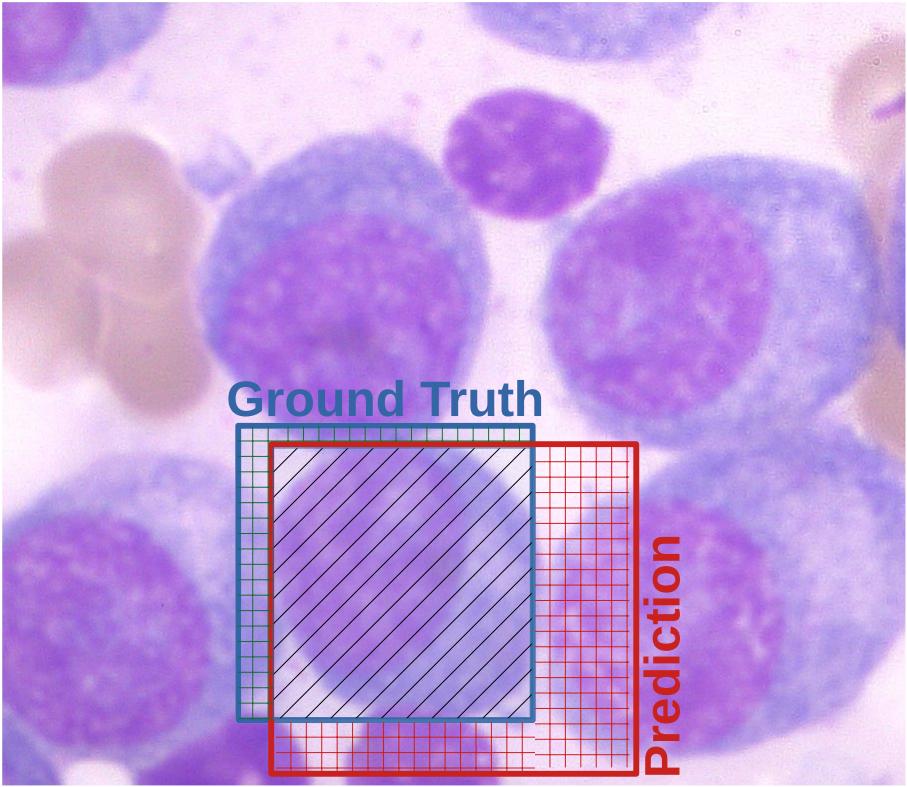
Bounding boxes illustrating a Region of Interest (ROI) defined by specialists (ground truth) in blue and a classification output in red. The black-hatched region constitutes the intersection area between both bounding boxes. The union area stands for all parts combining the colored boxes.

The evaluation methodology employed in this study adheres to the conventional practices of the Computer Vision field for object detection tasks. In summary, the detection performance is evaluated by comparing the areas between expected (a.k.a. ground truth) and predicted bounding boxes, as illustrated by the blue and red regions in Fig. 4, respectively.

Aiming to understand this evaluation better, consider the bounding box highlighted by a blue rectangle surrounding a plasma cell, which was defined by the specialists and is used to represent the ground truth area (G). The red bounding box illustrates the predicted area (P) detected by the DNN model. By considering these areas, we have used the Intersection Over Union (IOU) ratio as defined in Eq. 1 to estimate the numbers of true positives (TP), false positives (FP), and false negatives (FN). This estimation is based on a threshold, which works as a confidence interval to accept or reject the prediction. In a nutshell, if $\hbox {IOU} \ge \tau$, there is enough overlapping between the predicted and ground truth area to classify the detected object as true positive (TP). If the predicted area has no enough overlapped area with ground truth, a false positive (FP) is detected. Similarly, when a ground truth area is not overlapped by a prediction bounding box, it is considered a false negative (FN).

1 $$\begin{aligned} \hbox {IOU} = \frac{G \cap P}{G \cup P} \end{aligned}$$

Based on IOU, it is also possible to assess the model confidence (*C*), which can be used to measure the performance of predicting an object in a given bounding box. In summary, this confidence is calculated by the product between the probability of the presence of an object and IoU, such as $C = {\text {Pr}}({\text {Object}})*{\text {IOU}}$.

**Figure 5:**
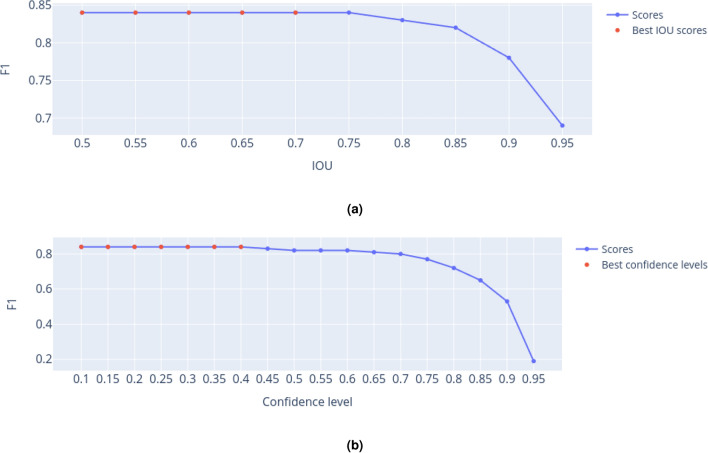
Results obtained by executing the grid search strategy to find (**a**) the best IOU threshold and (**b**) model confidence. The best results are represented by red dots.

Both the threshold ($\tau$) and the model confidence (*C*) are hyperparameters (not adjustable during the training phase), which can be varied to find objects in images better. Once there is no well-defined approach to set their values, we used a grid search in our experiments and selected their best combination based on the performance measured with F1-score, which is detailed next. In Fig. 5a, we show the results for $\tau \in [0.50, 0.95]$. One may notice that optimal performance is consistently attained when employing any of the first five options. Throughout our analysis, we have considered the default value (0.65) specified by the authors.

Similarly, we analyzed the model confidence across different values within the interval $C \in [0.10, 0.95]$, as shown in Fig. 5b. In this case, the first seven options yielded identical highest performances. In our experiments targeting plasma cell detection, we selected the default confidence value of 0.25, a recommendation also endorsed by the authors.

After calculating IOU, we are able to extract three significant metrics widely used in ML to assess the cell detection: Precision, Recall, F1-score, and precision-recall curve. Precision and Recall are calculated by using Eqs. 2 and 3, respectively. In summary, Precision assesses the models’ exactitude, i.e., it computes the rate of correct classifications for the positive label over the number of outcomes classified as positives. On the other hand, Recall measures the models’ completeness, which is calculated by the rate of correct classifications for the positive label over the number of elements expected to be under the positive label. We also evaluated the performance of our model using the F1-score metric that works as a harmonic mean of precision and recall, as shown in Eq. 4. The Precision-Recall curve allows to graphically visualize the general performance after several executions on different test folds.

2 $$\begin{aligned}{} & {} {\text {Precision}} = \frac{\hbox {TP}}{\hbox {TP} + \hbox {FP}} \end{aligned}$$

3 $$\begin{aligned}{} & {} {\text {Recall}} = \frac{\hbox {TP}}{\hbox {TP} + \hbox {FN}} \end{aligned}$$

4 $$\begin{aligned}{} & {} {\text {F1-score}} = \frac{2\times {\text {Precision}} \times {\text {Recall}}}{{\text {Precision}} + {\text {Recall}}} \end{aligned}$$

### Ethical approval

Project approved by the Institutional Research Committee - CEP ICS (CAAE - 70193723.5.0000.5662). The request for waiver of signing the TCLE to the CEP was justified by the use of slides and data tabulated and coded by specialists in the diagnosis of multiple myeloma in the clinical care hematological and laboratorial sectors of health care at a public university. This request was to encourage the procedure, since the evidence comes from sets of bone marrow aspirate slides from the bank of coded slides and integrated into the collections of MM diagnoses under the custody of hematologists and technicians from the diagnostic service at Hospital and Laboratory. This request by the Investigators avoids substantial risks to privacy and breach of trust between the physician, patient and investigator-researcher.

## Data Availability

The source code and datasets used in the study are freely available at https://github.com/LabIA-UFBA/Multiple-Myeloma-Dataset.
